# Strategies to overcome barriers to implementing osteoporosis and fracture prevention guidelines in long-term care: a qualitative analysis of action plans suggested by front line staff in Ontario, Canada

**DOI:** 10.1186/s12877-015-0099-8

**Published:** 2015-08-01

**Authors:** Sultan H. Alamri, Courtney C. Kennedy, Sharon Marr, Lynne Lohfeld, Carly J. Skidmore, Alexandra Papaioannou

**Affiliations:** Department of Medicine, Division of Geriatrics, McMaster University, 88 Maplewood Ave, Hamilton, ON L8M 1 W9 Canada; Juravinski Research Centre, Hamilton Health Sciences - St. Peter’s Hospital, 88 Maplewood Avenue, Hamilton, ON L8M 1 W9 Canada; Department of Clinical Epidemiology & Biostatistics, McMaster University, 1280 Main St. West, Hamilton, ON L8S4K1 Canada; Faculty of Medicine, King Abdulaziz University, Jeddah, Kingdom of Saudi Arabia; Geriatrics Residency Program, St. Joseph’s Hospital, McMaster University, 50 Charlton Ave. E., Hamilton, ON L8N 4A6 Canada

**Keywords:** Barriers, Osteoporosis, Fractures, Prevention, Qualitative

## Abstract

**Background:**

Osteoporosis is a major global health problem, especially among long-term care (LTC) facilities. Despite the availability of effective clinical guidelines to prevent osteoporosis and bone fractures, few LTC homes actually adhere to these practical recommendations. The purpose of this study was to identify barriers to the implementation of evidence-based practices for osteoporosis and fracture prevention in LTC facilities and elicit practical strategies to address these barriers.

**Methods:**

We performed a qualitative analysis of action plans formulated by Professional Advisory Committee (PAC) teams at 12 LTC homes in the intervention arm of the Vitamin D and Osteoporosis Study (ViDOS) in Ontario, Canada. PAC teams were comprised of medical directors, administrators, directors of care, pharmacists, dietitians, and other staff. Thematic content analysis was performed to identify the key themes emerging from the action plans.

**Results:**

LTC teams identified several barriers, including lack of educational information and resources prior to the ViDOS intervention, difficulty obtaining required patient information for fracture risk assessment, and inconsistent prescribing of vitamin D and calcium at the time of admission. The most frequently suggested recommendations was to establish and adhere to standard admission orders regarding vitamin D, calcium, and osteoporosis therapies, improve the use of electronic medical records for osteoporosis and fracture risk assessment, and require bone health as a topic at quarterly reviews and multidisciplinary conferences.

**Conclusions:**

This qualitative study identified several important barriers and practical recommendations for improving the implementation of osteoporosis and fracture prevention guidelines in LTC settings.

## Background

Osteoporosis is a major public health problem, affecting more than 200 million people worldwide [1]. In Canada, it is highly prevalent among long-term care (LTC) populations with a reported prevalence up to 86 % [2]. Fractures are the most serious complication of osteoporosis and comprise a significant cause of morbidity, mortality and burden to society [3]. In 2010, Osteoporosis Canada published the second evidence-based Clinical Practice Guidelines (CPGs) For The Diagnosis And Management Of Osteoporosis In Canada with a focus on preventing fragility fractures [4]. The guidelines recommend lifestyle modifications, smoking cessation, falls prevention strategies and adequate total (dietary and supplemental) calcium and vitamin D intake as well as antiresorptive therapy in high risk patients [4]. Despite the effectiveness of these interventions in preventing fragility fractures, they are underutilized and the majority of elderly persons residing in LTC facilities receive suboptimal osteoporosis care [5, 6]. This gap between what should be practiced according to clinical evidence and what is actually practiced is one of the most consistent findings in health care service research [7]. An in depth understanding of the barriers and facilitating factors responsible for such gap is important to improve adherence to CPGs in the LTC sector and to optimize the clinical care of elderly residents [8–10]. Furthermore, tailoring strategies to overcome identified barriers is more likely to enhance professionals’ clinical behavior [11].

The objectives of this paper were 1) to identify potential barriers to evidence-based practices for osteoporosis and fracture prevention in LTC settings and 2) to provide practical strategies to address those barriers based on a qualitative review of action plans made by members of Professional Advisory Committee (PAC) teams in LTC facilities, whose participation has been shown to increase the likelihood of guideline adoption [12].

## Methods

### Setting and participants

This qualitative study was conducted in twelve LTC homes in Ontario, Canada. All homes were active participants in the intervention arm of the Vitamin D and Osteoporosis Study (ViDOS). Briefly, ViDOS was a cluster, randomized controlled trial in 40 LTC homes (21 control; 19 intervention) that examined the feasibility and effectiveness of a knowledge translation intervention targeting integration of best practices for osteoporosis and fracture prevention. At each home, the target participants were the Professional Advisory Committee (PAC), an interdisciplinary team that meets quarterly to address resident care and quality improvement objectives. This group includes the medical director, director of care, administrator, consultant pharmacist, food services director, and other medical, nursing, and rehabilitation representatives.

### ViDOS intervention

Details of the study protocol have been published previously [13]. Over twelve-months, each intervention home participated in three interactive educational meetings (at months 1, 6 and 12) that included a standardized presentation and a question and answer session facilitated by a ViDOS expert opinion leader who was a specialist physician with expertise in osteoporosis or geriatrics. ViDOS experts engaged with study participants either in-person (meeting one only) or remotely with the study coordinator on-site to distribute/collect study materials. Facility-level audit and feedback reports for vitamin D, calcium, and osteoporosis medication prescribing, benchmarked against other ViDOS intervention homes, were presented in a graphical format at each meeting. Confidential, individual audit and feedback reports were also provided to each physician. Additional point of care tools distributed included process checklists and treatment alerts (a paper-based tool for consultant pharmacists to alert physicians about residents at increased fracture risk).

After the expert presentation, PAC teams engaged in action planning for quality improvement based on the plan-do-study-act (PDSA) cycle [14] using the brainstorming technique. Between educational meetings, teams worked on implementing action plans and progress was reviewed at the next meeting. Control homes received tool-kits provided to all Ontario LTC homes (www.osteoporosislongtermcare.ca) [15].

### Data collection and outcomes

Study outcomes in the ViDOS trial included both feasibility (e.g., recruitment, retention, data collection, and intervention fidelity) [16] and clinical outcomes (proportion of residents prescribed vitamin D, calcium, and osteoporosis medications) [17].

In this qualitative study, we focus only on the analysis of the action planning component of the ViDOS intervention. Using a brainstorming technique, PAC teams worked through the action plan work sheets considering 1) the potential barriers to implementing osteoporosis and fracture prevention guidelines and 2) the strategies that needed to be taken to overcome those barriers (What has to happen? Who should be involved? What do you need? What are your next steps?). The ViDOS coordinator assisted in capturing the information on the work sheets and provided updated versions.

### Data analysis

The team analyzed the action plans produced by each PAC team using Thematic Framework Analysis, a commonly used approach to combine deductive (theme-driven) and inductive (ad hoc) coding and analysis of qualitative data [18]. Two investigators (CCK, SHA) independently reviewed the action plans to develop an initial codebook. Then the investigators met with a team member with expertise in qualitative research (LL) to develop a comprehensive list of main themes. They then examined the themes in relation to the three types of barriers to implementing change in clinical care identified by Grol: individual, organization and social [19]. Finding a good degree of fit between the data and the model, the team decided to adopt the theoretical framework. The two analysts then mapped the contents of each action plan against the themes and presented the results to the larger team for discussion. Differences of opinion on how to categorize specific action plan items were resolved through group discussion until consensus was reached.

### Ethical considerations

This research project was reviewed and approved by the Hamilton Integrated Research Ethics Board (HIREB). Written informed consent was obtained from each participant prior to data collection.

## Results

### Characteristics of participating sites

Twelve LTC facilities across Ontario, Canada participated in the study with a mean bed size of 114 (SD: 57.0). LTC homes were located in communities that ranged in size from <30,000 people (42 %) to >1,000,000 people (17 %). The majority of homes were characterized as for-profit (92 %) and 75 % were affiliated with multi-facility chain operators.

### Barriers

The most commonly reported barriers to providing optimal bone health care in LTC were lack of information and educational resources prior to the ViDOS intervention, difficulty obtaining required patient information for fracture risk assessment and inconsistent prescribing of vitamin D and calcium at the time of admission. Moreover, failure to include osteoporosis and fracture prevention strategies as topics for quarterly reviews and the patient/family out-of-pocket cost of vitamin D were perceived as important obstacles. The full set of barriers is reported in Table 1.

**Table 1:** Barriers to the implementation of osteoporosis and fracture prevention guidelines in long-term care facilities

Category	Themes	Barriers
Individual	• Knowledge	• Patients have impaired understanding of their condition.
		• Staff members incorrectly dispense bisphosphonates.
	• Habits	• Few staff members attend educational sessions.
Organization	• Regulations	• Vitamin D and calcium are inconsistently prescribed at the time of admission^a^.
		• Osteoporosis and fracture prevention strategies are not discussed during multidspilinary conferences and quarterly reviews^a^.
		• Information required for fracture risk assessment is difficult to obtain^a^.
	• Processes of care	• There is a limited medical history regarding osteoporosis and fractures available to LTC facility.
		• The LTC facility does not examine changes in height on an annual basis.
		• The recommendation of three servings of dairy per day is not followed.
	• Resources	• Lack of information and educational resources prior to the ViDOS intervention^a^
Social	• Authorities	• The process to change policies is cumbersome.
	• Patient reactions	• The Patient or his/her family is not willing or unable to pay for vitamin D^a^.

^a^Reported by >60 % of the 12 participating homes

### Suggested strategies

The most frequently suggested strategies by the PAC teams were implementation of standard admission orders regarding vitamin D, calcium, and osteoporosis therapies; better use of electronic medical records (EMRs) for osteoporosis and fracture risk assessment; and addition of bone health care as a topic in quarterly reviews and multidisciplinary conferences. They also suggested using educational toolkits and videos for LTC staff, residents, and their families as well as greater input by dietitians for review and monitoring of calcium intake and to ensure adequate amounts of dietary calcium for LTC residents. The full set of strategies is reported in Table 2.

**Table 2:** Strategies to overcome barriers to the implementation of osteoporosis and fracture prevention guidelines in long-term care facilities

Category	Themes	Strategies
Individual	• Knowledge	• Educate patients and families about osteoporosis and fracture prevention in addition to various methods to conserve bone mass and reduce falls risk.
		• Improve awareness among health care professionals about bone health.
Organization	• Regulations	• Establish and adhere to standard admission orders regarding vitamin D, calcium, and osteoporosis therapies^a^.
		• Include bone health care in quarterly reviews and multidisciplinary conferences agendas^a^.
	• Processes of care	• Histories of osteoporosis, fractures, and other diagnoses must be properly documented in electronic medical records^a^.
		• The risks for osteoporosis and fractures must be regularly assessed at the time of admission.
		• The risk of falling must be regularly assessed.
		• Calcium must be incorporated into the diet.
	• Resources	• Regular educational sessions must be offered to long-term care staff using on-site experts, videos and toolkits^a,b^.
		• The availability of physicians must be increased.
		• A dietitian must be present to review and monitor calcium intake^a^.
		• Osteoporosis experts are required to provide example standard orders for long-term care facilities.
Social	• Patient reactions	• Fees related to the dispensing of vitamin D and calcium must be reduced.

^a^Reported by >60 % of the 12 participating homes

^b^Fracture prevention tool-kits were sent to all Ontario long-term care facilities by the Ontario Osteoporosis Strategy for Long-term care (www.osteoporosislongtermcare.ca) [15]

## Discussion

In this study, PAC teams from 12 LTC facilities in Canada identified barriers to osteoporosis care in LTC facilities and suggested strategies to overcome them. One of the most frequently reported barriers to the implementation of osteoporosis CPGs was the lack of information and resources prior to intervention educational meetings. Participants identified improved education of staff as well as patients and families as strategies to overcome such barriers. In particular, the use of videos and toolkits to educate staff members about bone health care was frequently suggested. Similar approaches have been shown to modestly improve osteoporosis management by health care providers [20]. Combined with other strategies (e.g., assessing osteoporosis/fracture risk on admission and including bone health in quarterly reviews), this approach may be useful in changing the low prioritization of osteoporosis and resistance to change among providers, both of which were previously identified factors in suboptimal osteoporosis care [11, 21].

Difficulty in obtaining necessary patient information (medical history, information about osteoporosis and fracture diagnoses, and annual height assessments) was another barrier reported by participants in our study. This finding is consistent with the results of a recent survey conducted with Ontario LTC Physicians (*n* = 87), who reported that barriers to the use of CPGs included lack of access to medical history and test results (e.g., bone mineral density) [6]. These physicians suggested removing bone mineral density from the fracture risk assessment, as supported by Rodondi et al. [22] who demonstrated that age and clinical risk factors were more important than bone mineral density in the calculation of 10-year fracture probability in nursing home residents. Improved use of EMRs was suggested by our study participants as a strategy to overcome this lack of information. Studies have shown that using EMR systems can improve the efficiency, quality, and accuracy of documentation and is supported by professional organizations such as the American Medical Directors Association and the American Health Care Association [23]. However, knowledge and competence in the use of these tools vary widely among facilities, indicating the need for education and training of LTC facility administrators and staff [24, 25].

Inconsistent prescribing of vitamin D and calcium on admission was also identified as a barrier to guideline adherence. Similarly, Teng et al. [21] reported that the lack of standard orders was an important barrier, concluding that system-wide intervention holds the most promise for overcoming barriers to high-quality osteoporosis care. Modification to delivery care systems was also reported to prevent fractures in LTC homes [26]. The suggestion by our study participants to establish standard orders for vitamin D, calcium, and osteoporosis therapies is one important strategy that may reduce fragility fractures within this setting. In addition, the French Group of Geriatrics and Nutrition has recommended systematic supplementation of vitamin D for all residents in LTC facilities and further suggests that intermittent (e.g., weekly) rather than daily supplementation may improve compliance [27]. This approach could potentially overcome patient reluctance to pay for vitamin D therapy, another potential barrier in our study.

### Limitations

Although we found brainstorming a useful technique for exploring barriers and solutions, combining multiple qualitative methods (literature search, surveys, focus groups or individual interviews) might have resulted in more data [28]. However, the time required to gather and analyze such data was not available in the larger study. Other limitations of this study include a relatively small sample size (12 LTC homes) and overrepresentation of for-profit homes (92 %), which may limit the ability to generalize the findings to non-profit facilities.

## Conclusion

This qualitative study outlined several important barriers to the implementation of evidence-based practices for osteoporosis and fracture prevention in LTC settings. Although tailoring strategies to overcome barriers needs to consider local context, the participants identified several common practical strategies at multiple levels that are relatively easy to implement and have been successfully implemented in the majority of LTC homes that participated in this study [16].

## Abbreviations

LTC: Long-term care
PAC: Professional Advisory Committee
ViDOS: Vitamin D and Osteoporosis Study
CPGs: Clinical practice guidelines
EMRs: Electronic medical records
